# Neuronal p58^IPK^ Protects Retinal Ganglion Cells Independently of Macrophage/Microglia Activation in Ocular Hypertension

**DOI:** 10.3390/cells12121558

**Published:** 2023-06-06

**Authors:** Todd McLaughlin, Jinli Wang, Liyun Jia, Fuguo Wu, Yaqin Wang, Joshua J. Wang, Xiuqian Mu, Sarah X. Zhang

**Affiliations:** 1 Department of Ophthalmology and Ross Eye Institute, Jacobs School of Medicine and Biomedical Sciences, University at Buffalo, State University of New York, Buffalo, NY 14203, USA; 2 Beijing Tongren Hospital, Capital Medical University, Beijing 100005, China; 3 Taihe Hospital, Hubei University of Medicine, Shiyan 442005, China; 4 Department of Biochemistry, University at Buffalo, State University of New York, Buffalo, NY 14203, USA

**Keywords:** p58^IPK^, retinal ganglion cells, glaucoma, macrophages, neurodegeneration

## Abstract

p58^IPK^ is a multifaceted endoplasmic reticulum (ER) chaperone and a regulator of eIF2α kinases involved in a wide range of cellular processes including protein synthesis, ER stress response, and macrophage-mediated inflammation. Systemic deletion of p58^IPK^ leads to age-related loss of retinal ganglion cells (RGC) and exacerbates RGC damage induced by ischemia/reperfusion and increased intraocular pressure (IOP), suggesting a protective role of p58^IPK^ in the retina. However, the mechanisms remain elusive. Herein, we investigated the cellular mechanisms underlying the neuroprotection action of p58^IPK^ using conditional knockout (cKO) mouse lines where p58^IPK^ is deleted in retinal neurons (Chx10-p58^IPK^ cKO) or in myeloid cells (Lyz2-p58^IPK^ cKO). In addition, we overexpressed p58^IPK^ by adeno-associated virus (AAV) in the retina to examine the effect of p58^IPK^ on RGC survival after ocular hypertension (OHT) in wild type (WT) mice. Our results show that overexpression of p58^IPK^ by AAV significantly improved RGC survival after OHT in WT mice, suggesting a protective effect of p58^IPK^ on reducing RGC injury. Conditional knockout of p58^IPK^ in retinal neurons or in myeloid cells did not alter retinal structure or cellular composition. However, a significant reduction in the b wave of light-adapted electroretinogram (ERG) was observed in Chx10-p58^IPK^ cKO mice. Deletion of p58^IPK^ in retinal neurons exacerbates RGC loss at 14 days after OHT. In contrast, deficiency of p58^IPK^ in myeloid cells increased the microglia/macrophage activation but had no effect on RGC loss. We conclude that deletion of p58^IPK^ in macrophages increases their activation, but does not influence RGC survival. These results suggest that the neuroprotective action of p58^IPK^ is mediated by its expression in retinal neurons, but not in macrophages. Therefore, targeting p58^IPK^ specifically in retinal neurons is a promising approach for the treatment of neurodegenerative retinal diseases including glaucoma.

## 1. Introduction

Progressive loss of functional retinal ganglion cells (RGCs) and their axons is considered the major and ultimate cause of visual impairment in glaucoma [1]. Current clinical treatment for glaucoma focuses on pharmacological, laser, or surgical therapies to lower intraocular pressure (IOP). While successful reduction in IOP could slow down the disease progression to some extent, in many patients it appears to be insufficient to prevent RGC loss and maintain normal visual function [2,3]. Among the currently identified pathological pathways, systemic and local inflammation has been linked to retinal neurodegeneration in glaucoma [4]. Enhanced inflammatory responses, mediated by activated glial cells including astrocytes, Müller cells, and microglia, and blood-derived immune cells such as monocytes/macrophages and T cells, have been observed before RGC death in glaucomatous retinas; however, their exact implications and regulations during RGC injury and disease progression remain undefined [4,5]. Understanding the complex molecular basis of the degenerative process of the retina and the RGCs may identify new therapeutic targets for the treatment of retinal disease. 

The unfolded protein response (UPR) is a highly conserved stress response mechanism that plays a critical role in maintaining cellular homeostasis and function in the central nervous system (CNS) neurons [6]. The UPR is activated in stress conditions when excessive unfolded or misfolded proteins accumulate in the endoplasmic reticulum (ER). A key element of the UPR is to increase the protein folding capacity by upregulation of molecular chaperones such as p58^IPK^, a member of the heat shock protein (HSP) 40 family encoded by the DNAJC3 gene [7,8]. Unlike classical ER chaperones, p58^IPK^ is believed to possess a dual topology either existing as a free luminal floating protein in the ER or residing at the ER membrane with the C-terminus facing the cytosol [9]. In the ER lumen, p58^IPK^ can bind to unfolded proteins and the major ER chaperone glucose regulated protein 78 (GRP78) promoting protein folding and homeostasis [10]. When localized to the ER membrane, p58^IPK^ functions as an inhibitor of the eIF2α kinases, such as protein kinase double-stranded RNA-dependent (PKR), PKR-like ER kinase (PERK), and general control non-derepressible-2 (GCN2), and regulates protein translation by reducing eIF2α phosphorylation [8,11,12]. In macrophages, p58^IPK^ was found to inhibit PKR-dependent inflammasome activation, TNF-α and IL-6 expression, and IL-1β production, suggesting an immune regulatory function of p58^IPK^ [13]. These multifaceted functions of p58^IPK^ in the regulation of protein homeostasis and inflammatory response make it an attractive target in research concerning neurodegenerative disease of the retina. 

Because the pathogenesis of glaucoma is not fully understood and multiple pathways have been implicated in neurodegeneration, a therapeutic intervention at the critical, irreversible stage of RGC death is of interest. Previously, we demonstrated that p58^IPK^ is highly expressed in retinal neurons including RGCs and global p58^IPK^ knockout mice exhibit increased RGC loss during aging and in animal models of neurotoxin-induced retinal degeneration, retinal ischemia/reperfusion (I/R), and ocular hypertension (OHT) [14,15]. In contrast, overexpression of p58^IPK^ significantly reduces cell death in cultured retinal cells including primary RGCs under oxidative or ER stress conditions [14,15]. This suggests that p58^IPK^ can protect the neural retina and RGCs from stress induced cell loss. However, it is unclear whether the protective effect of p58^IPK^ is attributable to the intrinsic signaling in retinal neurons and/or its anti-inflammatory effect in immune cells that indirectly affects RGC survival. Herein, we investigate the cell-specific role of p58^IPK^ in retinal neurons and in macrophages under normal and glaucomatous stress conditions taking advantage of two newly generated p58^IPK^ conditional knockout (cKO) mouse lines. Our results suggest that the neuroprotective effect of p58^IPK^ in OHT is mediated by its intrinsic role in retinal neurons but not its expression in macrophages. In addition, targeting RGCs directly with a neuroprotective agent will preserve vision regardless of disease pathophysiology.

## 2. Materials and Methods

### 2.1. Animals

The p58^IPK^ flox mouse line was generated using a targeting construct generated by inserting a loxP site upstream of the p58^IPK^ transcription start site and a loxP-FRT-Neo-FRT cassette in the first exon of P58^IPK^. This construct was then used to electroporate G4 129xC57BL/6 F1 hybrid ES cells [16]. After selection by G418 and ganciclovir, positive clones were identified by Southern blot hybridization with a 5′ and 3′ external probe, in which the wild type allele and correctly targeted allele yielded 19-kb and 10.5- and 7.5-kb fragments, respectively. Two positive clones were then injected into C57/BL6 blastocysts to generate chimeric mice. High percentage chimeric males were crossed with wild type C57BL/6 females for germ-line transmission. Progeny were genotyped by PCR for the presence of loxP and Neomycin. Positive mice were then bred with the FLPeR mice to delete the Neo cassette and generate P58^IPK^ flox mice. The primers for genotyping P58^IPK^ flox were: 5′-TAAAGGCACTGGAAGCATTT-3′ and 5′-AAGTAGGAACTATCTTCACC-3. The LysMCre line has been previously published [17] and was purchased from Jackson Labs (strain 004781; B6.129P2-*Lyz2^tm1(cre)Ifo^*/J). The Chx10-cre line has been described elsewhere [18] and successfully used in our previous study [19]. Lysozyme M (LysM), encoded by the Lyz2 gene, is exclusively expressed in myelomonoytic cells including monocytes, macrophages and granulocytes in mice. By crossing homozygous p58^IPK^ floxed (fl) mice to the LysMCre line we generated p58^IPK^ fl/fl; LysMCre + (Lyz2-p58^IPK^ cKO) mice, which lack p58^IPK^ specifically in macrophages [17]. Similarly, by crossing p58^IPK^ floxed mice to the Chx10-Cre line we generated p58^IPK^ fl/fl; Chx10-Cre + (Chx10-p58^IPK^ cKO) mice, in which p58^IPK^ is deleted in the vast majority of retinal neurons and a subset of Müller glia [20]. The p58^IPK^ flox/flox mice were backcrossed to a C57/BL6J background and crossed with LysMCre or Chx10-cre mice to generate p58^IPK^ flox/flox; LysMCre/+ (Lyz2-p58^IPK^ cKO) or p58^IPK^ flox/flox; Chx10-cre (Chx10-p58^IPK^ cKO) for experiments. Both Lyz2-p58^IPK^ cKO and Chx10-p58^IPK^ cKO lines were also crossed with the mTmG reporter line (Jackson Labs, strain 007676; B6.129(Cg)-Gt(ROSA)26Sortm4(ACTB-tdTomato,-EGFP)Luo/J), in which all cells constitutively express Tomato (red fluorescent protein) unless a cell expresses Cre recombinase, in which case Tomato production permanently ceases in those cells and constitutive green fluorescent protein (GFP) production begins [21]. The absence of Rd1 and Rd8 mutations was confirmed by genotyping. All experiments were performed in compliance with protocols approved by the Institutional Animal Care and Use Committees at the University at Buffalo.

### 2.2. Microbead-Induced OHT Model

The microbead induced OHT model has a long history and broad acceptance as a glaucoma model across species including mice through the induction the dysfunction of the outflow facility of aqueous humor [18]. Microbead injection was performed as described previously [15,22]. Briefly, mice were anesthetized using ketamine, and the xylazine and pupils were dilated with sequential application of 2.5% phenylephrine hydrochloride ophthalmic solution (Akorn) and 1% atropine sulfate ophthalmic solution (Falcon). The anterior chamber was injected with 2 µL PBS containing 10µm diameter polystyrene, fluorescent microspheres at 1.5 × 10^7^ beads/mL (FluoSpheres, Invitrogen, Eugene, OR, USA using a Hamilton syringe fitted with a 33g needle. IOP was recorded every other day, at the same time of day, by bounce tonometer (Icare, Vantaa, Finland). Mice that developed ocular edema, hemorrhage, cataracts, or that failed to sustain OHT were excluded from the study.

### 2.3. Immunohistochemistry and Analyses

Mouse eye cryosections were prepared for immunohistochemistry (IHC) as described previously [19]. Briefly, eyes were removed immediately after sacrifice and fixed for 1 h with 4% paraformaldehyde in PBS, then washed in PBS. For cryosectioning, eyes were equilibrated in 30% sucrose in PBS, embedded in O.C.T. compound (Fisher, Houston, TX, USA) and frozen. Cryosections at 20 µm thickness were cut and dried. For retinal wholemount IHC, retinas were dissected and retinal cups processed through the IHC protocol. Retina sections or retinal cups were blocked in PBS plus 1% Triton X-100 plus 1% BSA for 1 h. Primary antibody was applied at the appropriate concentration in block solution overnight at 4 °C (with shaking for wholemounts). Tissue was washed in block solution 3–5 times for 5–10 min then incubated in secondary antibody diluted to the appropriate concentration in block solution for 1 h. Tissue was washed and mounted for photography directly (wholemounts) or mounted in VectaShield with DAPI (Vector Laboratories, Burlingame, CA, USA, cryosections). The primary antibodies used were: Ribeye (Synaptic Systems, Göttingen, Germany, 192003, 1:800); PKC-α (Santa Cruz, Dallas, TX, USA, sc-8393, 1:600); GABA (MilliporeSigma, Burlington, MA, USA, PC213L, 1:800); SCGN (BioVendor, Asheville, NC, USA, RD184120100); Iba1 (Wako, Richmond, VA, USA, 019-19741, 1:800); GFAP (Dako, 30334, 1:800); Cone Arrestin (MilliporeSigma, AB15282, 1:1000). The following secondary antibodies were all used at 1:800 dilution: Texas-red conjugated goat-anti-rabbit (ThermoFisher, Waltham, MA, USA, T6391); Alexa 594 conjugated goat-anti-mouse (ThermoFisher, A11005); Alexa 594 conjugated rabbit-anti-goat (ThermoFisher, A11080); Alexa 488 conjugated goat-anti-mouse (ThermoFisher, A11001); Alexa 488 conjugated donkey-anti-rabbit (ThermoFisher, A21206); Alexa 568 conjugated donkey-anti-goat (ThermoFisher, A11057); Alexa 488 conjugated donkey-anti-sheep (ThermoFisher, A11015); Alexa 488 conjugated goat-anti-rabbit (ThermoFisher, A11034).

All analyses were performed blind to condition and genotype. After wholemount staining for Brn3a, retinas were flat-mounted by making 3–5 radial relief cuts and mounting in PBS with ganglion cell layer facing up. Four images per retina were taken at a position 50–70% radially from the optic disc to the periphery and absent major blood vessels. Cell counts per animal were averaged and n values represent number of mice. Images for retinal wholemount with Iba1 labeling were obtained similarly, with care taken to identify the appropriate focal planes for superficial, mid-, and deep-retinal layers. Cryosections were analyzed in regions approximately 400–900 µm from the optic nerve head and normalized per 100 µm linear distance, where appropriate. Between three and six areas across at least two sections were anlayzed per mouse. Cell counts were averaged per animal and n values represent number of mice.

### 2.4. Dark- and Light-Adapted Electroretinography (ERG)

Electroretinogram (ERG) were performed on the Diagnosys Espion ColorDome system and manufacturer software (Diagnosys LLC, Lowell, MA, USA) essentially as previously described [19]. Briefly, mice were anesthetized with 100 mg/kg ketamine and 7.5 mg/kg xylazine. Pupils were dilated with 1% atropine (Falcon Pharmaceuticals, Fort Worth, TX, USA) followed by 2.5% Phenylephrine Hydrochloride (Bausch & Lomb, Portland, OR, USA). A ground electrode was inserted into the tail and a second electrode placed subcutaneously centrally between the eyes as reference. Ophthalmic gel (Gonak, Akorn, Lake Forest, IL, USA) was applied to the cornea and electrodes placed in contact with the gel across each cornea. For light-adapted ERGs, the stimulation was five flashes of 4 ms duration at 1 Hz at 10 cd s/m^2^ with a background of 5 cd s/m^2^. 

Dark-adapted ERGs were performed using the protocol previously described [19]. Briefly, mice were dark adapted overnight and then anesthetized and dilated as described above. A stimulus of ten series of three flashes of light of 4 ms duration was applied with a delay between each series of flashes of 15–60 s (delay period increases with stimulus intensity). The amplitude for the a wave was the lowest point of the initial response relative to baseline. The b wave amplitude was calculated relative to the a wave.

### 2.5. Isolation and Culture of Primary Mouse BMDMs

BMDMs were isolated and cultured as previously described [13,20]. After 7 days of culturing in DMEM/F12 containing 10% FBS, penicillin, streptomycin, and 50 ng/mL macrophage-colony stimulating factor (M-CSF, Peprotech, Cranbury, NJ, USA), 0.6 million BMDMs were seeded in each well of the 12 well plate. Cells were then harvested for either quantitative RT-PCR or Western blot analysis.

### 2.6. RNA Isolation and Quantitative Real-Time PCR (qPCR)

RNA was isolated from mouse retinas or BMDMs using Trizol following the manufacturers protocol (Invitrogen, Waltham, MA, USA). RNA was converted into cDNA using the iScript cDNA synthesis kit (Bio-Rad, Hercules, CA, USA). The expression of p58^IPK^ was measured by qPCR using the SYBR green supermix (Bio-Rad) and CFX96 Touch Real-Time PCR System (Bio-Rad). The primers for qPCR were: p58^IPK^ forward: 5′- TCCTGGTGGACCTGCAGTACG-3′ and reverse: 5′-CTGCGAGTAATTTCTTCCCC-3′; 18S ribosomal RNA forward: 5′-GTAACCCGTTGAACCCCATT-3′ and reverse: 5′-CCATCCAATCGGTAGTAGCG-3′.

### 2.7. Western Blot Analysis

Mouse retinal tissue or BMDMs were lysed with radio immune precipitation assay (RIPA) buffer with a protease inhibitor mixture, PMSF, and sodium orthovanadate (Santa Cruz Biotechnology). Protein concentration was quantified using the Pierce^TM^ BCA protein assay kit (ThermoFisher). Samples were loaded into a 10% SDS-PAGE gel, and transferred to nitrocellulose membrane for immunoblotting using anti-p58^IPK^ (Cell Signaling Technology, Danvers, MA, USA, C56E7, 1:1000) and anti-β-actin (Abcam, Cambridge, United Kingdom, ab8226, 1:10000) antibody. After incubation with HRP-conjugated secondary antibodies, membranes were developed with Clarity and Clarity Max ECL Western Blotting Substrate (Bio-Rad, CA, USA) using Chemi-Doc MP Imaging System (Bio-Rad, CA, USA). The bands were semi-quantified by densitometry using Image J software.

### 2.8. Intravitreal Injection of AAVs

Mice were anesthetized with 100 mg/kg ketamine and 5 mg/kg xylazine. Both pupils were dilated with 1% atropine (Falcon Pharmaceuticals) followed by 2.5% Phenylephrine Hydrochloride (Bausch & Lomb). Either 1.5 µL of AAV-GFP or AAV-p58^IPK^-GFP [15] at equivalent titers of 5 × 10^12^ GC/mL were injected into the vitreous with a Hamilton syringe fitted with a 33G needle. Ophthalmic gel (Gonak, Akorn) was applied to the cornea immediately after injection.

### 2.9. Statistical Data Analysis

Data are shown as mean ± SD. Statistical analysis was performed using GraphPad Prism 9 (GraphPad Software Inc., La Jolla, CA, USA). Students’ t-test was used in the comparison of two groups and one-way ANOVA was used for comparisons between three or more groups. A result of a *p*-value of less than 0.05 was considered significant.

## 3. Results

### 3.1. In Vivo Overexpression of p58IPK by AAV in the Retina Reduces RGC Loss in OHT

**Figure 1:**
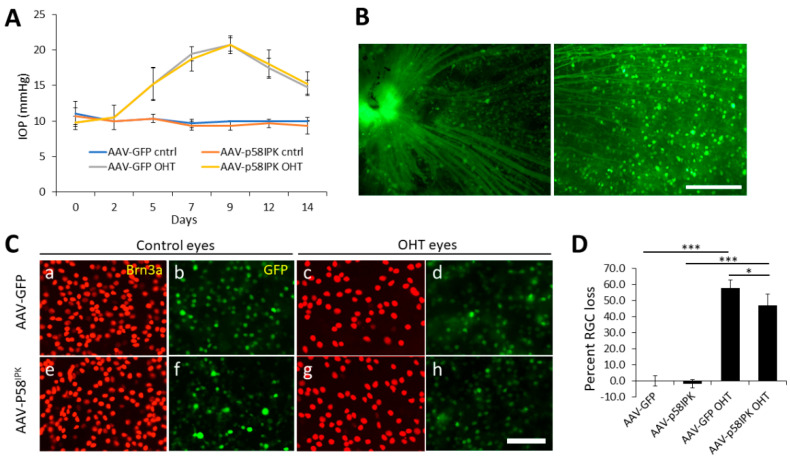
**In vivo overexpression of p58^IPK^ in retina reduces RGC loss in an ocular hypertension model.** Wild type mice were intravitreally injected in one eye with adeno-associated virus expressing GFP (AAV-GFP) and intravitreally injected in the contralateral eye with AAV expressing p58^IPK^ (AAV-p58^IPK^). (**A**) Graph depicts average intraocular pressure (IOP) as recorded in the eyes after microbead-induced ocular hypertension (OHT) and in the contralateral sham control eyes on alternate days for two weeks. There are no differences in IOP between AAV-GFP and AAV-p58^IPK^ injected eyes in either group. (**B**) Representative images of retinal wholemounts showing GFP expression in the soma and axons of RGCs after AAV transduction. Scale bar = 150 µm. (**C**) Photomicrographs of wholemount immunohistochemistry with antibodies against Brn3a (red; a, c, e, g) to label RGCs and GFP expression in the same focal plane from AAV-GFP (b, d) and AAV-p58^IPK^ (f, h) to show the representative extent of viral infections. Scale bar = 50 µm. (**D**) Graph depicts the loss of Brn3a+ retinal ganglion cells (RGCs) in OHT eyes treated with AAV-GFP or AAV-p58^IPK^ compared to AAV-treated sham control eyes. Results show that there is a significant decrease in RGC loss (47%) in AAV-p58^IPK^-treated OHT eyes compared to AAV-GFP-treated OHT eyes (n = 3–4). *, *p* < 0.05; ***, *p* < 0.001.

Previously we demonstrated that overexpression of p58^IPK^ reduces apoptosis and increases survival of cultured R28 retinal progenitor cells and primary mouse RGCs under induced ER stress and oxidative stress conditions [14,15]. To determine if the neuroprotective effect of p58^IPK^ operates in vivo, we overexpressed p58^IPK^ in the retina by an intravitreal injection of AAVs expressing p58^IPK^ (AAV-p58^IPK^) and a green fluorescent protein (GFP) into one eye in adult C57/BL6J mice. The contralateral eye of each mouse received AAVs expressing GFP (AAV-GFP) as control. Two weeks after the injections, the mice were randomly assigned to receive a microbeads injection to induce OHT (experimental group) or a sham procedure (control group) in both eyes. As shown in Figure 1A, IOP was increased in the experimental group from day 5 to day 14 after microbeads injection identically in AAV-p58^IPK^-treated and AAV-GFP-treated eyes (Figure 1A). Successful transduction by AAVs is demonstrated by GFP expression in the soma and axons of RGCs (Figure 1B). Two weeks after the induction of OHT, Brn3a-postive RGCs were quantified in retinal wholemounts as described in Materials and Methods. In the control group, no difference was observed in the number of Brn3a-positive RGCs between eyes treated with AAV-p58^IPK^ or AAV-GFP, indicating that RGC survival is not affected by AAV-p58^IPK^ under normal IOP conditions (Figure 1C,D). In the experimental group, AAV-p58^IPK^-treated eyes showed a 47% loss of Brn3a-positive RGCs, which was significantly lower than AAV-GFP-treated eyes with a 58% loss of RGCs (Figure 1C,D). These results suggest that overexpression of p58^IPK^ in retina mitigates the glaucomatous loss of RGCs in a model of OHT.

### 3.2. Generation of Retina-Specific and Myeloid Cell-Specific p58IPK cKO Mouse Lines

Our prior work shows that global knockout of p58^IPK^ augments RGC loss during aging and in several disease models including OHT and I/R [14,15]. We have also demonstrated that p58^IPK^ suppresses PKR signaling and reduces inflammatory cytokine production from macrophages [13]. To delineate whether the neuroprotective role of p58^IPK^ in the retina is attributed to its intrinsic signaling in retinal neurons or due to its anti-inflammatory function in macrophages, we generated two novel cKO mouse lines in which p58^IPK^ is deleted specifically in retinal progenitor cells or in myeloid cells (see Experimental Procedures). No gross abnormalities in growth, body weight, or blood glucose were observed in the floxed or cKO lines. 

The fidelity of cre expression in each cKO line was verified by crossing Lyz2-p58^IPK^ cKO mice and Chx10-p58^IPK^ cKO mice with mTmG mice, a double-fluorescent Cre reporter line which constitutively expresses a conditional tdTomato transgene in the absence of Cre recombinase but switches to expressing GFP upon exposure to Cre recombinase [17]. As shown in Supplementary Figure S1, GFP expression was observed in macrophages from the myeloid lineage present in the choroid (Supplementary Figure S1A–C) and the spleen (Supplementary Figure S1D–F) in the Lyz2-p58^IPK^ cKO line. In Chx10-p58^IPK^ cKO mice the cre expression was found in the vast majority of cells throughout the retinal wholemounts (Supplementary Figure S1G,H). 

**Figure 2:**
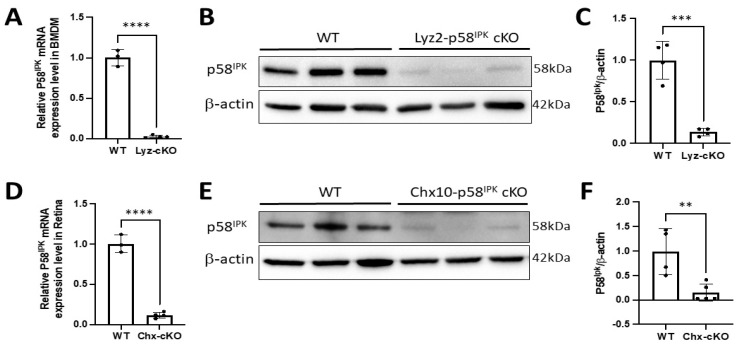
High knockout efficiency of p58^IPK^ in macrophages of Lyz2-p58^IPK^ cKO mice and in the retina of Chx10-p58^IPK^ cKO mice. Quantitative RT-PCR (**A**,**D**) and Western blot (**B**,**C**,**E**,**F**) analysis were carried out using RNA and protein extracted from bone marrow derived macrophages (BMDMs, **A**–**C**) or retinas (**D**–**F**) of WT and p58^IPK^ cKO mice. (**A**) There is a significant (97%) reduction in p58^IPK^ mRNA in BMDMs derived from Lyz2-p58^IPK^ cKO mice (n = 4) compared to WT (n = 3). (**B**,**C**) p58^IPK^ protein level is reduced by 86% in BMDMs derived from Lyz2-p58^IPK^ cKO mice (n = 4) compared to WT (n = 4). (**D**) qPCR reveals a 90% reduction in p58^IPK^ mRNA in the retina of Chx10-p58^IPK^ cKO mice (n = 4) compared to WT (n = 3). (**E**,**F**) Western blot analysis shows a significant 85% reduction in p58^IPK^ protein level in the retina of Chx10-p58^IPK^ cKO mice (n = 5) compared to WT (n = 4). **, *p* < 0.01; ***, *p* < 0.001; ****, *p* < 0.0001.

To determine the Cre-mediated knockout efficiency, we isolated bone marrow-derived macrophages (BMDMs) from Lyz2-p58^IPK^ cKO and p58^IPK^ fl/fl (WT) mice. RNA expression and protein level of p58^IPK^ were determined by qRT-PCR and Western blotting, respectively. We find a reduction of 97% of p58^IPK^ mRNA and an 86% reduction in p58^IPK^ protein in BMDMs derived from Lyz2-p58^IPK^ cKO mice compared to WT controls (Figure 2A–C). Similarly, the retinas from Chx10-p58^IPK^ cKO showed a 90% reduction of p58^IPK^ mRNA and an 85% reduction in p58^IPK^ protein compared to WT retina (Figure 2D–F). These results confirm a robust KO efficiency mediated by Cre expression in macrophages of Lyz2-p58^IPK^ cKO mice and in retinal cells of Chx10-p58^IPK^ cKO mice. 

### 3.3. Impacts of p58^IPK^ Deficiency in Macrophages or Retinal Neurons on Retinal Structure or Function

**Figure 3:**
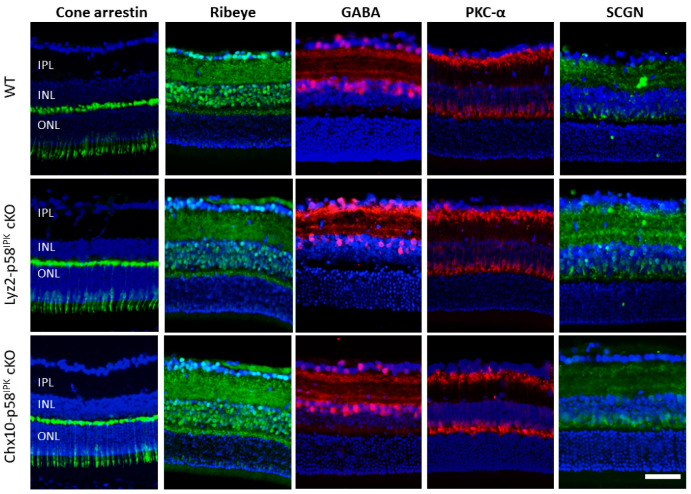
**Immunohistochemical analysis of retinal neuronal markers in Lyz2-p58^IPK^ and Chx10-p58^IPK^ cKO mice.** Representative images of immunofluorescence staining for retinal neuronal markers for cone photoreceptors (cone arrestin), synapses between photoreceptors and bipolar cells (Ribeye), GABAergic amacrine cells (GABA), rod bipolar cells (PKC-α), and cone bipolar cells (SCGN) in retinal cryosections from WT, Lyz2-p58^IPK^ cKO and Chx10-p58^IPK^ cKO mice. Nuclei were stained with DAPI (blue). Scale bar = 50 µm.

We examined retinal structure and neuronal populations by immunohistochemistry in adult Lyz2-p58^IPK^ cKO mice and Chx10-p58^IPK^ cKO mice under normal, non-diseased conditions. We find that the expression pattern and intensity of markers for multiple retinal cell types as well as synaptic markers are indistinguishable in the retinas from both p58^IPK^ cKO lines and WT controls (Figure 3). Specifically, there are no apparent morphological differences in cone photoreceptors (labeled with cone arrestin), rod bipolar cells (labeled with PKC-α), cone bipolar cells (labeled with secretagogin, SCGN), and GABAergic amacrine cells (labeled with GABA) between WT and p58^IPK^ cKO mice (Figure 3). In addition, ribbon synapses in the outer plexiform layer (OPL) are indistinguishable in both p58^IPK^ cKO lines compared to WT (Figure 3). 

**Figure 4:**
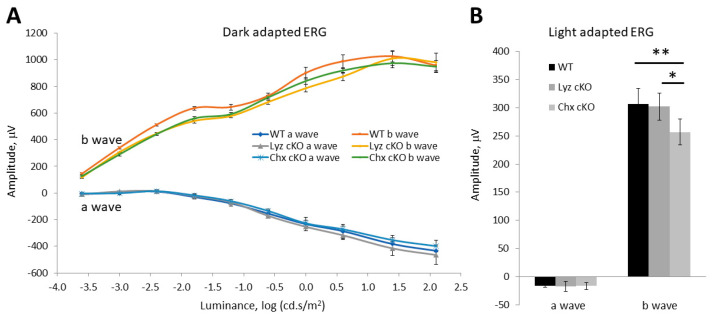
**Reduced retinal function in Chx10-p58^IPK^ cKO mice.** (**A**) Graph depicts dark-adapted ERG a wave and b wave responses across 10 steps of increasing stimulus luminance for wild type (WT; n = 11), Lyz2-p58^IPK^ cKO (n = 5), and Chx10-p58^IPK^ cKO mice (n = 5). (**B**) Graph depicts the a wave and b wave responses of a transient, light-adapted ERG for WT (n = 8), Lyz2-p58^IPK^ cKO (n = 6), and Chx10-p58^IPK^ cKO (n = 6) mice. There is no difference in a wave response between WT and Lyz2-p58^IPK^ cKO mice. There is a significant decrease in b wave response in Chx10-p58^IPK^ cKO mice compared to WT and Lyz2-p58^IPK^ cKO mice. All values are mean ± S.D. *, *p* < 0.05; **, *p* < 0.01.

To assess the impact of p58^IPK^ deficiency on retinal function, we performed light- and dark-adapted electroretinogram (ERG) on adult WT mice and p58^IPK^ cKO mice. In dark-adapted (scotopic) ERG, we found no significant differences in a wave or b wave responses in Lyz2-p58^IPK^ cKO mice nor in Chx10-p58^IPK^ mice compared to WT (Figure 4A). However, the b wave amplitude in the light-adapted (photopic) ERG was significantly reduced in Chx10-p58^IPK^ cKO mice compared to WT and Lyz2-p58^IPK^ cKO mice by 16.2% and 15.1%, respectively (Figure 4B). No difference was identified between Lyz2-p58^IPK^ cKO mice and WT. These results verify that the Lyz2-p58^IPK^ cKO retina is essentially indistinguishable from WT in the context of retinal structure and function under normal conditions. In contrast, the b wave reduction observed in Chx10-p58^IPK^ cKO mice indicates a possible dysfunction of bipolar cells and/or of the synapses between bipolar cells and photoreceptors, which warrants further investigation.

### 3.4. Increased RGC Loss after OHT in Chx10-p58^IPK^ cKO Mice, but Not in Lyz2-p58^IPK^ cKO Mice

**Figure 5:**
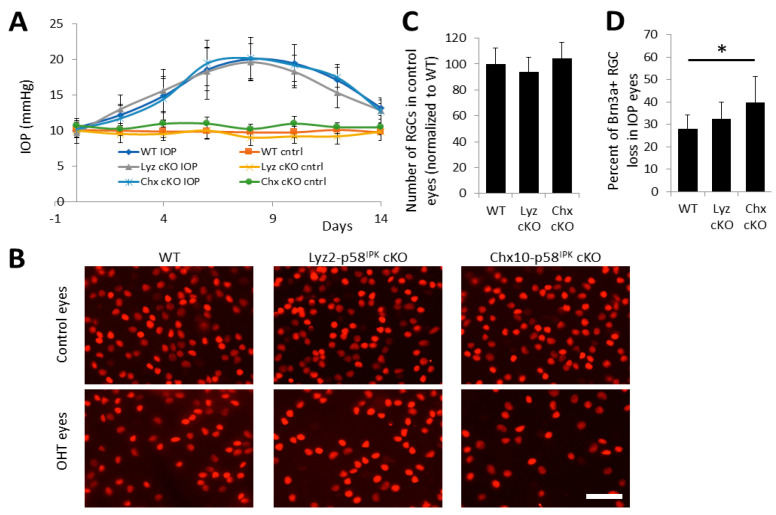
**Increased RGC loss in Chx10-p58^IPK^ cKO mice after ocular hypertension.** Wild type (WT, n = 10), Lyz2-p58^IPK^ cKO (n = 6), and Chx10-p58^IPK^ cKO (n = 4) mice were subjected to microbead-induced OHT. (**A**) IOP was monitored every other day after induction of OHT for two weeks. There are no differences in IOP for control eyes or for OHT eyes between any groups. (**B**) Representative images of retinal wholemounts immunostained with an antibody against Brn3a. (**C**) Graph depicts a similar number of Brn3a-positive RGCs in WT, Lyz2-p58^IPK^ cKO, and Chx10-p58^IPK^ cKO in control eyes. (**D**) Graphs depicts a significant increase in RGC loss after OHT in Chx10-p58^IPK^ cKO mice compared to WT. Scale bar = 50 µm. *, *p* < 0.05.

To determine the cell-specific role of p58^IPK^ in RGC survival in glaucomatous conditions, we induced OHT in both p58^IPK^ cKO lines and WT mice. In this scenario, one eye in each animal is mock-injected and serves as sham control and the other eye is subjected to an injection of microbeads into the anterior chamber to induce an increase in IOP. All three strains respond similarly to microbead injection and have similar increases in IOP (Figure 5A). In control eyes, we find no statistically significant difference in the number of Brn3a-positive RGCs between strains (Figure 5B,C). However, in eyes subjected to OHT, we find a significant increase in the loss of Brn3a-positive RGCs in Chx10-p58^IPK^ cKO mice (40% RGC loss) when compared to WT mice (28% RGC loss), whereas the RGC loss was not significantly altered in Lyz2-p58^IPK^ cKO eyes (33% RGC loss) compared to WT (Figure 5B,D). These results suggest that p58^IPK^ in retinal neurons, but not in macrophages, is required for its neuroprotective effect in RGCs in the OHT model.

### 3.5. Increased Macrophage/Microglial Activation in the Retina of Lyz2-p58^IPK^ cKO Mice after OHT

**Figure 6:**
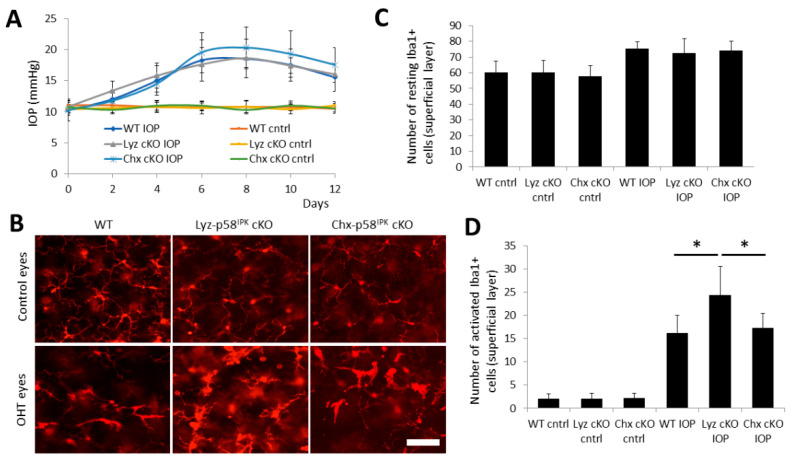
**Increased macrophage/microglia activation in the superficial layer of the retina in Lyz2-p58^IPK^ cKO mice after OHT.** Wild type (WT, n = 10), Lyz2-p58^IPK^ cKO (n = 6), and Chx10-p58^IPK^ cKO (n = 4) mice were subjected to microbead-induced OHT. (**A**) IOP was monitored every other day after induction of OHT for two weeks. There are no differences in IOP between control eyes or in OHT eyes between any strains. (**B**) Representative images of retinal wholemounts immunostained with anti-Iba1 antibody showing activated Iba1-positive macrophages/microglia in the superficial layer of the retina in OHT eyes. Scale bar = 50 µm. (**C**) Graph depicts a small, though not significant increase in the numbers of resting Iba1+ cells in OHT eyes compared to control eyes in WT, Lyz2-p58^IPK^ cKO, and Chx10-p58^IPK^ cKO mice. (**D**) Graph depicts a significant increase in activated Iba1+ cells in OHT eyes in all three strains of mice. In addition, there is a significant increase in activated Iba1+ cells in Lyz2-p58^IPK^ cKO retinas compared to WT or Chx10-p58^IPK^ cKO retinas after OHT. *, *p* < 0.05.

Next, we determine if the changes in RGC loss are associated with activation of macrophage/microglia in the retina of p58^IPK^ cKO after OHT. We confirmed no difference in IOPs among the groups of cKO and WT mice (Figure 6A). Two weeks after OHT, retinal wholemounts were obtained and stained with an anti-Iba-1 antibody to label macrophages and microglia (Figure 6B). The number, morphology, and location of Iba-1-positive cells were analyzed. In sham control eyes, no difference was observed in the numbers of resting (ramified cells with highly branched processes) or activated (larger and wider cell body with retracted and fewer processes) Iba1-positive cells in the superficial layer of WT, Lyz2-p58^IPK^ cKO, and Chx10-p58^IPK^ cKO retinas (Figure 6C,D). Compared to control eyes, there was a small increase in the total number of resting Iba1-positive cells in the superficial layer of the retina in OHT eyes, with no difference between WT, Lyz2-p58^IPK^ cKO, and Chx10-p58^IPK^ cKO retinas (Figure 6C). Notably, there was a greater than 7-fold increase in the numbers of activated Iba1-positive cells in the superficial layer of the retina in OHT eyes compared to control eyes for all strains (Figure 6D). In addition, there was a significant increase in activated Iba1-positive cells in the superficial retinal layer of the OHT eyes in Lyz2-p58^IPK^ cKO mice by 50.9% and 40.7% compared to WT and Chx10-p58^IPK^ cKO mice, respectively (Figure 6D). In deeper retinal layers, we find no differences between the three strains in the number and activation state of Iba1-positive cells in control eyes or OHT eyes (Supplementary Figure S2). These data indicate that loss of p58^IPK^ in macrophages results in a greater extent of macrophage/microglia activation in a mouse OHT model.

**Figure 7:**
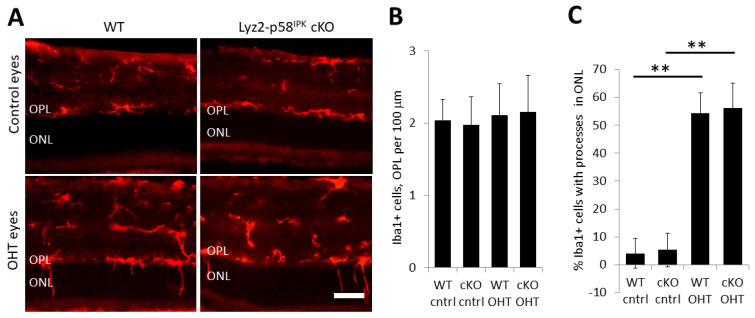
**Increased extension of Iba1+ cell processes into the ONL in OHT eyes.** Retinal cryosections from wild type (WT, n = 3) and Lyz2-p58^IPK^ cKO (cKO, n = 4) mice at two weeks after OHT were immunostained for Iba1. (**A**) Representative images of Iba1 staining reveal extension of the processes of Iba1-positive macrophages/microglia into the outer nuclear layer (ONL) in OHT eyes. Scale bar = 50 µm. (**B**) Graph depicts no difference in the number of Iba1+ cells in the outer plexiform layer (OPL). (**C**) Graph depicts a significant increase in the proportion of Iba1+ cells with processes extending into the ONL in OHT eyes in both WT and Lyz2-p58^IPK^ cKO mice. There is no difference between WT and Lyz2-p58^IPK^ cKO in this measure. **, *p* < 0.01.

**Figure 8:**
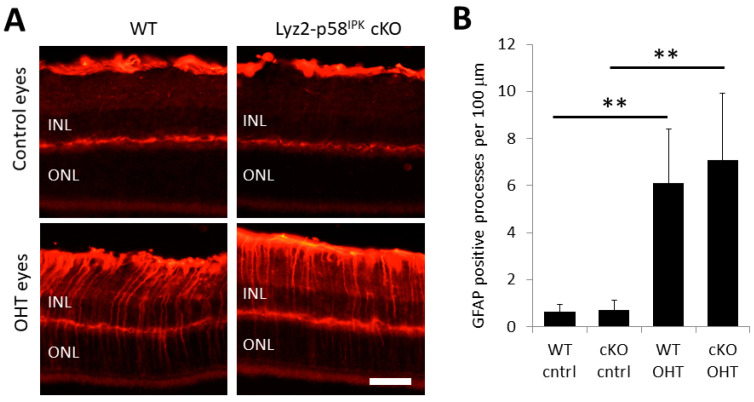
**Increased Müller cell activation in OHT eyes.** Retinal cryosections from wild type (WT, n = 3) and Lyz2-p58^IPK^ cKO (cKO, n = 4) mice at two weeks after OHT were immunostained for GFAP. (**A**) Representative images of GFAP staining showing activation of Müller cells with processes extending across all retinal layers, including the inner nuclear layer (INL) and outer nuclear layer (ONL) in OHT eyes. Scale bar = 50 µm. (**B**) Graph depicts a significant increase in the number of GFAP-positive processes in OHT eyes but no difference in this increase between WT and Lyz2-p58^IPK^ cKO mice. **, *p* < 0.01.

We further investigated macrophage/microglia activation in the OPL using immunohistochemistry on retinal cryosections of control retinas and retinas from Lyz2-p58^IPK^ cKO and WT mice (Figure 7A). After two weeks of OHT, we found no increase in the total number of Iba1-positive cells residing in the OPL in WT or Lyz2-p58^IPK^ cKO retinas (Figure 7B). Interestingly we found a significant increase in the number of Iba1-positive cells in the OPL with extended processes into the ONL in both WT and Lyz2-p58^IPK^ cKO mice, though this increase is not significantly different between the groups (Figure 7C). We further examined the activation of Müller cells by immunostaining for GFAP in the retinas of WT and Lyz2-p58^IPK^ cKO mice. We found that there was a significant increase in GFAP-positive Müller cell processes in the retinas of both WT and Lyz2-p58^IPK^ cKO mice subjected to increased IOP, compared to control eyes (Figure 8). However, we observed no significant difference between WT and Lyz2-p58^IPK^ cKO retinas, though there is a trend for an increased number in the Lyz2-p58^IPK^ cKO retinas. These findings suggest that the broad inflammatory response in the retina after OHT is not affected by the loss of p58^IPK^ in macrophages.

## 4. Discussion

In this study, we investigated the contribution of neuron-specific and myeloid cell-specific p58^IPK^ to RGC survival in normal and glaucomatous conditions. Using two newly generated cell type-specific p58^IPK^ cKO mouse lines, we show that deletion of p58^IPK^ in retinal neurons, but not in myeloid cells, exacerbated glaucomatous RGC loss in a microbead-induced OHT model. These results corroborate our prior work in global p58^IPK^ KO mice, which demonstrate that a lack of p58^IPK^ results in increased apoptosis and cell death of RGCs in multiple physiological and pathological conditions including aging, N-methyl-D-aspartic acid (NMDA)-induced neurotoxicity, retinal ischemia, increased IOP, and ER stress [14,15]. In addition, we have shown that overexpression of p58^IPK^ in the retina by AAV significantly reduced RGC loss after OHT, further confirming the findings from in vitro studies that p58^IPK^ overexpression improves retinal neuronal cell survival in cultured retinal R28 cells or primary RGCs challenged with ER stress or oxidative stress [14,15]. Collectively, our study supports a critical role of the intrinsic signaling of p58^IPK^ in protecting RGCs in chronic stress conditions pertinent to retinal diseases such as glaucoma.

p58^IPK^ was originally identified as a cellular regulator of PKR inhibiting the PKR’s activity in phosphorylating eIF2α and reducing protein synthesis during influenza virus infection [23]. Subsequent studies suggest that p58^IPK^ is a general inhibitor of eIF2α kinases (PKR, PERK, and GCN2), playing a critical role in controlling and fine-tuning the cellular stress response [8,11,12]. During ER stress, upregulation of p58^IPK^ suppresses the PERK-UPR pathway and defects in p58^IPK^ function lead to sustained PERK activation, reduced protein translation, and increased cell death [8,10]. In contrast, overexpression of cytoplasmic p58^IPK^ inhibits the activity of eIF2α kinases, such as GCN2, a stress response protein induced by amino acid deprivation to modulate protein translation [12]. Dysregulation of the balance and function of the p58^IPK^/eIF2α kinases pathway contributes to cell death in multiple stress conditions. Clinically, patients bearing loss-of-function mutations of DNAJC3 (p58^IPK^) suffer from diabetes and multisystemic neurodegeneration [24,25,26]. The diabetes-associated pathological and pathophysiological changes are recapitulated in global p58^IPK^ KO that homozygous deletion of p58^IPK^ leads to apoptosis of pancreatic islet cells and gradual onset of glucosuria and hyperglycemia [27,28]. Cultured pancreatic islets from patients carrying p58^IPK^ gene mutations also demonstrate impaired mitochondrial function, lipid accumulation, increased ER stress, and β cell apoptosis. These results suggest an important role of p58^IPK^ in β cell survival and function, in part through regulation of cellular stress response [27,29,30]. Future studies are needed to investigate the potential impact and mechanisms of p58^IPK^ on mitochondrial function and lipid metabolism in animal models.

In contrast to an extensively studied role in β cells, the implications of p58^IPK^ in the CNS neurons and neurodegenerative disease are less well defined, which is possibly due to the complex phenotype of global p58^IPK^ KO mice with systemic metabolic abnormalities as a confounding factor [27,28]. To address this challenge, we generated a mouse line with floxed alleles of p58^IPK^ that has allowed us to investigate the cell-specific role of p58^IPK^ in the retina under normal and stressed conditions. We have demonstrated that this conditional allele of p58^IPK^ is efficiently excised by two independent Cre lines, resulting in near complete loss of p58^IPK^ mRNA and protein in cells expressing Cre recombinase. Further, the loss of p58^IPK^ in Lyz2-cre-expressing myelomonocytic cells (monocytes, macrophages, and granulocytes) or in Chx10-cre-expressing retinal progenitor cells does not affect retinal morphology and distribution of major retinal neuronal populations. Interestingly, we find a functional deficiency in adult Chx10-p58^IPK^ cKO light adapted ERG b wave with no change in the a wave, indicating that p58^IPK^ deficiency leads to dysfunction of post-synaptic retinal cells to cone photoreceptors and/or disturbance in the synapses between cone cells and bipolar cells. Recent studies have shown that ATF6, which is a major transcriptional regulator of p58^IPK^, is required for cone development and function [31] and mutations of ATF6 cause achromatopsia, an autosomal recessive cone dysfunction disease [32]. The exact function of p58^IPK^ in post-synaptic retinal cells in the cone signaling, such as cone bipolar cells, Müller cells, or intrinsically photosensitive retinal ganglion cells (ipRGCs), and its implication in maintaining retinal circuitry warrant future investigation.

Extending our previous findings from in vitro studies using cultured primary RGCs or R28 cells [14,15], we demonstrate here that AAV-mediated overexpression of p58^IPK^ in retina reduces RGC loss in a microbead-induced OHT model, confirming a protective effect of p58^IPK^ on RGC survival in vivo. Notably, we observed a higher degree of RGC loss induced by OHT in AAV-transduced retinas (Figure 1) compared to the group of animals without AAV treatment (Figure 5). These results are in line with previous findings from another glaucoma-relevant model that AAV-transduced RGCs are more susceptible to injury and cell death after optic nerve crush (ONC) [33], although the underlying mechanisms remain elusive. Given the compelling evidence suggesting an involvement of neuroinflammation in glaucomatous RGC injury [34,35,36,37], it is important to delineate whether the neuroprotective effect of p58^IPK^ is attributed to its intrinsic signaling in retinal neurons and/or its anti-inflammatory role in macrophages. To this end, we subjected the Chx10-p58^IPK^ cKO and Lyz2-p58^IPK^ cKO mice, and controls, to OHT and myriad analyses. Our results indicate that knockout of p58^IPK^ in retinal neurons significantly increased RGC loss after OHT in Chx10-p58^IPK^ cKO compared to WT and Lyz2-p58^IPK^ cKO mice. In contrast, deletion of p58^IPK^ in myelomonocytic cells neither enhances RGC loss nor protects the retina from RGC loss, despite a significant increase in activated Iba1-positive cells in the superficial retina layer of Lyz2-p58^IPK^ cKO mice after OHT. This may indicate that p58^IPK^ deficiency does increase macrophage activation; however, the change is not sufficient to contribute to cell loss, or to their protection in disease. One caveat to this conclusion is that the time frame of analysis in our disease model is two weeks. It is feasible that the long-term effects of p58^IPK^-deficiency in macrophages would manifest in disease models or aging. Furthermore, macrophages and Müller cells communicate under disease conditions, and we find an increased inflammatory response in the OHT retina, as evinced by an increase in GFAP expression in Müller cells. Though we do not see a significant difference in GFAP between WT and Lyz2-p58^IPK^ cKO mice, there is a trend for higher GFAP expression in Lyz2-p58^IPK^ cKO. This may indicate that p58^IPK^ in macrophages has some role in macrophage-Müller communication in relation to inflammatory response in stressed retina, which should be further investigated at additional time points after OHT and in other disease models.

In summary, the results from the present study using cell-specific p58^IPK^ cKO mouse lines revealed an important role of p58^IPK^ as an intrinsic protective factor in retinal neurons that promotes RGC survival against glaucomatous damage. Deletion of p58^IPK^ in myelomonocytic cells increased macrophage/microglial activation in stressed neural retina but was insufficient to influence RGC survival. Overall, these findings suggest that targeting neuronal p58^IPK^ may provide a novel approach for neuroprotection and treatment in retinal disease.

## Data Availability

The datasets and materials used and/or analyzed during the current study are available from the corresponding author on reasonable request.

## Supplementary Materials

The following supporting information can be downloaded at: https://www.mdpi.com/article/10.3390/cells12121558/s1, Figure S1: Validation of cell-specific cre expression in Lyz2-p58IPK cKO and Chx10-p58IPK cKO lines. Figure S2: Effect of increased IOP on Iba1-positive cells in deep retinal layers in WT, Lyz2-p58^IPK^ cKO, and Chx10-p58^IPK^ cKO mice.

## Abbreviations

AAV: adeno-associated virus; BMDMs; bone marrow-derived macrophages; BSA: bovine serum albumin; cKO; conditional knockout; CNS: central nervous system; DAPI: 4’,6-diamidino-2-phenylindole; ER: endoplasmic reticulum; ERG: electroretinogram; GABA: gamma-aminobutyric acid; GCN2: general control non-derepressible-2; GFAP: glial fibrillary acidic protein; GFP: green fluorescent protein; GRP78: glucose regulated protein 78; hr: hour; HSP: heat shock protein; Iba1: ionized calcium-binding adapter molecule 1; IHC: immunohistochemistry; I/R: ischemia/reperfusion; IOP; intraocular pressure; KO: knockout; O.C.T.: optimal cutting temperature; OHT: ocular hypertension; PBS: phosphate buffered saline; PERK: PKR-like ER kinase; PKC-α: protein kinase C alpha; PKR; protein kinase double-stranded RNA-dependent; qPCR: quantitative real-time PCR; RGC: retinal ganglion cells; SCGN: secretagogin; UPR: unfolded protein response; WT: wild type.

## References

[1] Almasieh M., Levin L.A. (2017). Neuroprotection in Glaucoma: Animal Models and Clinical Trials. Annu. Rev. Vis. Sci..

[2] Storgaard L., Tran T.L., Freiberg J.C., Hauser A.S., Kolko M. (2021). Glaucoma Clinical Research: Trends in Treatment Strategies and Drug Development. Front. Med..

[3] Alqawlaq S., Flanagan J.G., Sivak J.M. (2019). All roads lead to glaucoma: Induced retinal injury cascades contribute to a common neurodegenerative outcome. Exp. Eye Res..

[4] Wei X., Cho K.S., Thee E.F., Jager M.J., Chen D.F. (2019). Neuroinflammation and microglia in glaucoma: Time for a paradigm shift. J. Neurosci. Res..

[5] Coyle S., Khan M.N., Chemaly M., Callaghan B., Doyle C., Willoughby C.E., Atkinson S.D., Gregory-Ksander M., McGilligan V. (2021). Targeting the NLRP3 Inflammasome in Glaucoma. Biomolecules.

[6] McLaughlin T., Medina A., Perkins J., Yera M., Wang J.J., Zhang S.X. (2022). Cellular stress signaling and the unfolded protein response in retinal degeneration: Mechanisms and therapeutic implications. Mol. Neurodegener..

[7] Van Huizen R., Martindale J.L., Gorospe M., Holbrook N.J. (2003). P58IPK, a Novel Endoplasmic Reticulum Stress-inducible Protein and Potential Negative Regulator of eIF2α Signaling. J. Biol. Chem..

[8] Yan W., Frank C.L., Korth M.J., Sopher B.L., Novoa I., Ron D., Katze M.G. (2002). Control of PERK eIF2α kinase activity by the endoplasmic reticulum stress-induced molecular chaperone P58IPK. Proc. Natl. Acad. Sci. USA.

[9] Daverkausen-Fischer L., Pröls F. (2021). Dual topology of co-chaperones at the membrane of the endoplasmic reticulum. Cell Death Discov..

[10] Rutkowski D.T., Kang S.-W., Goodman A.G., Garrison J.L., Taunton J., Katze M.G., Kaufman R.J., Hegde R.S., Gilmore R. (2007). The Role of p58IPK in Protecting the Stressed Endoplasmic Reticulum. Mol. Biol. Cell.

[11] Gale M., Tan S.L., Wambach M., Katze M.G. (1996). Interaction of the interferon-induced PKR protein kinase with inhibitory proteins P58IPK and vaccinia virus K3L is mediated by unique domains: Implications for kinase regulation. Mol. Cell. Biol..

[12] Roobol A., Roobol J., Bastide A., Knight J.R., Willis A.E., Smales C.M. (2015). p58IPK is an inhibitor of the eIF2alpha kinase GCN2 and its localization and expression underpin protein synthesis and ER processing capacity. Biochem. J..

[13] Boriushkin E., Wang J.J., Li J., Bhatta M., Zhang S.X. (2016). p58(IPK) suppresses NLRP3 inflammasome activation and IL-1beta production via inhibition of PKR in macrophages. Sci. Rep..

[14] Boriushkin E., Wang J.J., Li J., Jing G., Seigel G.M., Zhang S.X. (2015). Identification of p58IPK as a Novel Neuroprotective Factor for Retinal Neurons. Investig. Ophthalmol. Vis. Sci..

[15] McLaughlin T., Dhimal N., Li J., Wang J.J., Zhang S.X. (2018). p58IPK Is an Endogenous Neuroprotectant for Retinal Ganglion Cells. Front. Aging Neurosci..

[16] George S.H., Gertsenstein M., Vintersten K., Korets-Smith E., Murphy J., Stevens M.E., Haigh J.J., Nagy A. (2007). Developmental and adult phenotyping directly from mutant embryonic stem cells. Proc. Natl. Acad. Sci. USA.

[17] Clausen B.E., Burkhardt C., Reith W., Renkawitz R., Förster I. (1999). Conditional gene targeting in macrophages and granulocytes using LysMcre mice. Transgenic Res..

[18] Rowan S., Cepko C.L. (2004). Genetic analysis of the homeodomain transcription factor Chx10 in the retina using a novel multifunctional BAC transgenic mouse reporter. Dev. Biol..

[19] McLaughlin T., Falkowski M., Park J.W., Keegan S., Elliott M., Wang J.J., Zhang S.X. (2018). Loss of XBP1 accelerates age-related decline in retinal function and neurodegeneration. Mol. Neurodegener..

[20] Zhang X., Goncalves R., Mosser D.M. (2008). The isolation and characterization of murine macrophages. Curr. Protoc. Immunol..

[21] Muzumdar M.D., Tasic B., Miyamichi K., Li L., Luo L. (2007). A global double-fluorescent Cre reporter mouse. Genesis.

[22] Chen H., Wei X., Cho K.-S., Chen G., Sappington R., Calkins D.J., Chen D.F. (2011). Optic Neuropathy Due to Microbead-Induced Elevated Intraocular Pressure in the Mouse. Investig. Ophthalmol. Vis. Sci..

[23] Lee T.G., Tang N., Thompson S., Miller J., Katze M.G. (1994). The 58,000-dalton cellular inhibitor of the interferon-induced double-stranded RNA-activated protein kinase (PKR) is a member of the tetratricopeptide repeat family of proteins. Mol. Cell. Biol..

[24] Ocansey S., Pullen D., Atkinson P., Clarke A., Hadonou M., Crosby C., Short J., Lloyd I.C., Smedley D., Assunta A. (2022). Biallelic DNAJC3 variants in a neuroendocrine developmental disorder with insulin dysregulation. Clin. Dysmorphol..

[25] Synofzik M., Tobias H.B., Kopajtich R., Gorza M., Rapaport D., Greiner M., Schönfeld C., Freiberg C., Schorr S., Reinhard H.W. (2014). Absence of BiP Co-chaperone DNAJC3 Causes Diabetes Mellitus and Multisystemic Neurodegeneration. Am. J. Hum. Genet..

[26] Ozon Z.A., Alikasifoglu A., Kandemir N., Aydin B., Gonc E.N., Karaosmanoglu B., Celik N.B., Eroglu-Ertugrul N.G., Taskiran E.Z., Haliloglu G. (2020). Novel insights into diabetes mellitus due to DNAJC3-defect: Evolution of neurological and endocrine phenotype in the pediatric age group. Pediatr. Diabetes.

[27] Ladiges W.C., Knoblaugh S.E., Morton J.F., Korth M.J., Sopher B.L., Baskin C.R., MacAuley A., Goodman A.G., LeBoeuf R.C., Katze M.G. (2005). Pancreatic beta-cell failure and diabetes in mice with a deletion mutation of the endoplasmic reticulum molecular chaperone gene P58IPK. Diabetes.

[28] Han J., Song B., Kim J., Kodali V.K., Pottekat A., Wang M., Hassler J., Wang S., Pennathur S., Back S.H. (2015). Antioxidants Complement the Requirement for Protein Chaperone Function to Maintain β-Cell Function and Glucose Homeostasis. Diabetes.

[29] Lytrivi M., Senée V., Salpea P., Fantuzzi F., Philippi A., Abdulkarim B., Sawatani T., Marín-Cañas S., Pachera N., Degavre A. (2021). DNAJC3 deficiency induces β-cell mitochondrial apoptosis and causes syndromic young-onset diabetes. Eur. J. Endocrinol..

[30] Jennings M.J., Hathazi D., Nguyen C.D.L., Munro B., Münchberg U., Ahrends R., Schenck A., Eidhof I., Freier E., Synofzik M. (2021). Intracellular Lipid Accumulation and Mitochondrial Dysfunction Accompanies Endoplasmic Reticulum Stress Caused by Loss of the Co-chaperone DNAJC3. Front. Cell Dev. Biol..

[31] Kroeger H., Grandjean J.M.D., Chiang W.-C.J., Bindels D.D., Mastey R., Okalova J., Nguyen A., Powers E.T., Kelly J.W., Grimsey N.J. (2021). ATF6 is essential for human cone photoreceptor development. Proc. Natl. Acad. Sci. USA.

[32] Kohl S., Zobor D., Chiang W.C., Weisschuh N., Staller J., Menendez G.I., Chang S., Beck S.C., Garrido G.M., Sothilingam V. (2015). Mutations in the unfolded protein response regulator ATF6 cause the cone dysfunction disorder achromatopsia. Nat. Genet..

[33] Li Y., Struebing F.L., Wang J., King R., Geisert E.E. (2018). Different Effect of Sox11 in Retinal Ganglion Cells Survival and Axon Regeneration. Front. Genet..

[34] Pupo A., Fernández A., Low S.H., François A., Suárez-Amarán L., Samulski R.J. (2022). AAV vectors: The Rubik’s cube of human gene therapy. Mol. Ther..

[35] Williams P.A., Marsh-Armstrong N., Howell G.R. (2017). Neuroinflammation in glaucoma: A new opportunity. Exp. Eye Res..

[36] Russo R., Varano G.P., Adornetto A., Nucci C., Corasaniti M.T., Bagetta G., Morrone L.A. (2016). Retinal ganglion cell death in glaucoma: Exploring the role of neuroinflammation. Eur. J. Pharmacol..

[37] Tezel G. (2022). Molecular regulation of neuroinflammation in glaucoma: Current knowledge and the ongoing search for new treatment targets. Prog. Retin. Eye Res..

