# Supplementary material for: Neuronal p58IPK Protects Retinal Ganglion Cells Independently of Macrophage/Microglia Activation in Ocular Hypertension
Source: Cells. 2023 Jun 6;12(12):1558. doi: 10.3390/cells12121558 (PMC10297187; doi:10.3390/cells12121558)
Supplement: Supplementary file 1 [file cells-12-01558-s001.zip › cells-2142401-supplementary.pdf]

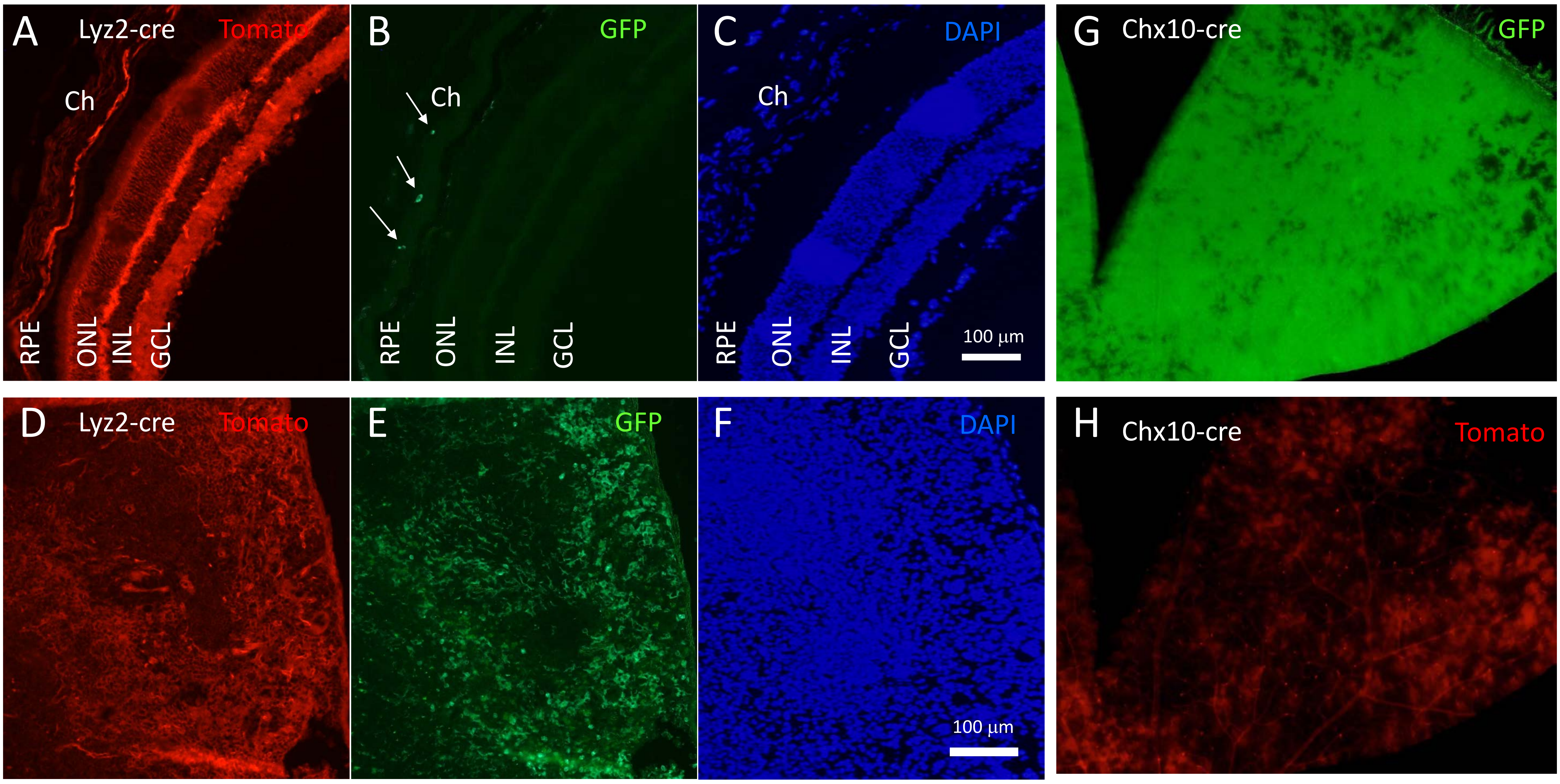

Supplemental Figure S1. Validation of cell-specific cre expression in Lyz2-p58IPK cKO and Chx10-p58IPK cKO lines. Both Lyz2 cKO-p58IPK and Chx10-p58IPK cKO mice lines were crossed with the mTmG reporter line in which Tomato (red) is constitutively expressed. When cre-recombinase is expressed, Tomato expression is halted, and GFP (green) expression begins. (**A-C**) Retinal sections from Lyz2-p58IPK cKO, mTmG mice show GFP expression in macrophages (arrows) in the choroid. (**D-F**) Robust GFP expression in macrophages in the spleen of Lyz2-p58IPK cKO, mTmG mice. (**G&H**) Retinal wholemounts from Chx10-p58IPK cKO, mTmG mice show GFP expression in vast majority of retinal cells throughout the retina, with a significant minority of Tomato expressing cells including vascular cells. Scale bar = 100 µm.

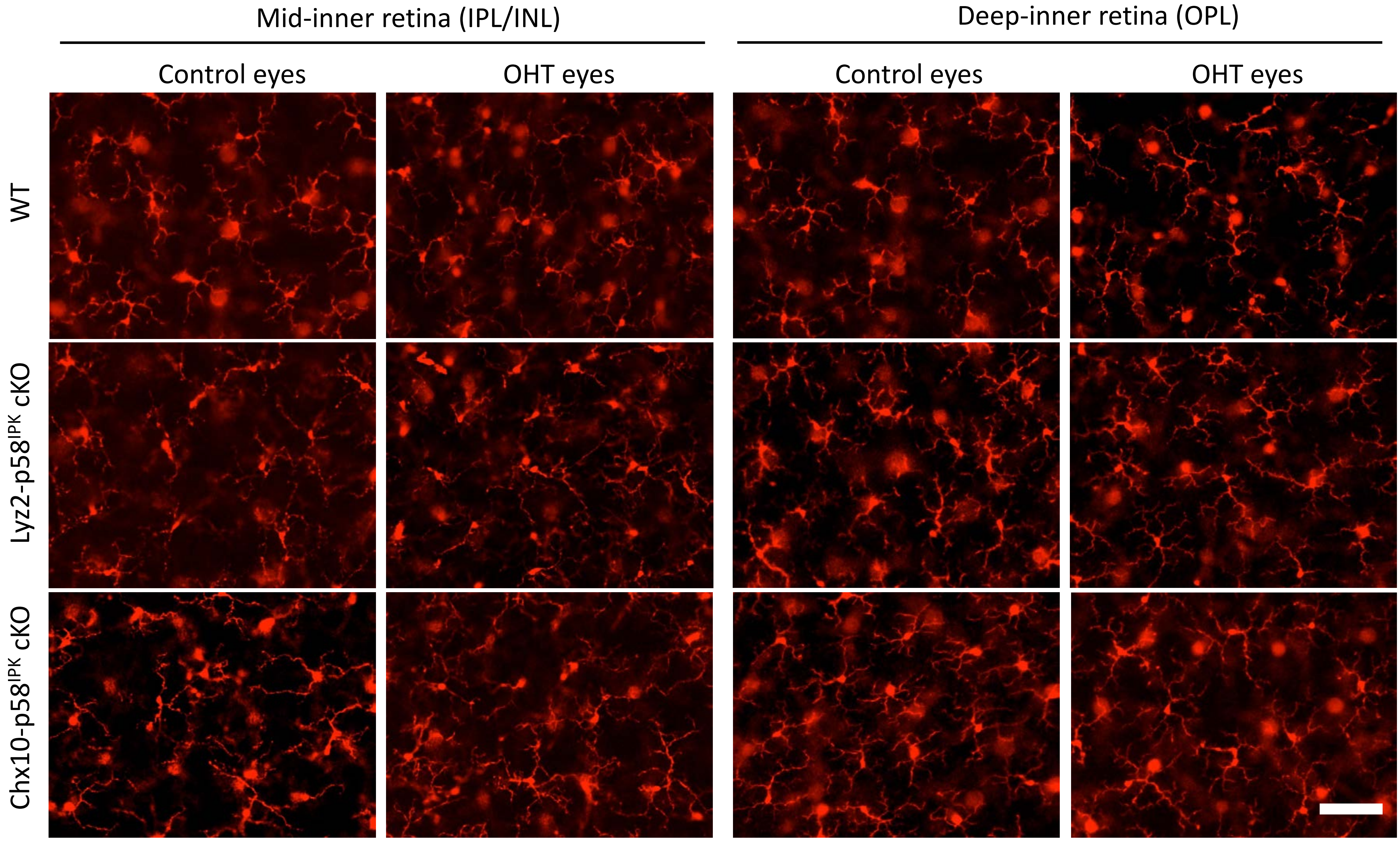

Supplemental Figure S2. Effect of increased IOP on Iba1-positive cells in deep retinal layers in WT, Lyz2-p58^{IPK} cKO, and Chx10-p58^{IPK} cKO mice. Representative images of retinal wholemounts with immunofluorescence staining for Iba1 in the inner plexiform layer/inner nuclear layer (IPL/INL) and outer plexiform layer (OPL). There is no apparent increase in the overall number of Iba1-positive cells (red), nor in the number of activated Iba1-positive cells, in OHT eyes versus control eyes. Scale bar = 50µm.
